# Fortification of Water Kefir with Gallic Acid: Physicochemical Effects and Antioxidant Capacity in an In Vitro Digestion Model

**DOI:** 10.3390/foods15111945

**Published:** 2026-06-01

**Authors:** Uriel Felipe Valdez-Olmos, José Carlos Rodríguez Figueroa, Ariadna Thalía Bernal-Mercado, Fridha Viridiana Villalpando-Vargas, Esther Sendra-Nadal, Carmen Lizette Del-Toro-Sánchez

**Affiliations:** 1 Departamento de Investigación y Posgrado en Alimentos, Universidad de Sonora, Encinas y Rosales s/n, Hermosillo 83000, Sonora, Mexico; a213212994@unison.mx (U.F.V.-O.); thalia.bernal@unison.mx (A.T.B.-M.); 2 Departamento de Ingeniería Química y Metalurgia, Universidad de Sonora, Encinas y Rosales s/n, Hermosillo 83000, Sonora, Mexico; 3 Departamento de Ciencias de la Salud, Centro Universitario de los Valles (CUVALLE), Universidad de Guadalajara, Carretera a Guadalajara Km. 45.5, Ameca 46600, Jalisco, Mexico; viridiana.villalpando@academicos.udg.mx; 4 Grupo de Investigación “Calidad y Seguridad Alimentaria”, Instituto de Investigación e Innovación Agroalimentaria y Agroambiental (CIAGRO-UMH), Universidad Miguel Hernández, Carretera de Beniel, Km 3.2, Orihuela, 03312 Alicante, Spain; esther.sendra@umh.es

**Keywords:** water kefir, gallic acid, antioxidant, fortification

## Abstract

Today, consumers are interested in healthier food. Fortifying water kefir (WK) with gallic acid (GA) could enhance kefir’s health benefits. The objective of this study was to evaluate the effects of GA-fortified WK (WK-GA) and pasteurization on physicochemical parameters and antioxidant capacity after an in vitro digestion. The results showed that WK-GA had significantly higher (*p* < 0.05) dry biomass than WK, 9.52 and 9.22 g/L, respectively. No significant differences were found (*p* > 0.05) at pH and °Brix kinetics. Antioxidant capacity kinetics using DPPH^•^ and ABTS^•+^ showed that WK-GA maintained its antioxidant effect throughout the fermentation process, inhibiting free radicals by 89.9% and 98.6%, respectively. In FRAP, WK-GA reached 14.63 mM TE/mL, while WK had 4.19 mM TE/mL. Furthermore, the antioxidant capacity of the bioavailable fractions decreased in DPPH^•^ and FRAP, even after pasteurization. However, in the ABTS^•+^ assay, the antioxidant capacity of the bioavailable fraction remained at 94% inhibition. These results demonstrate the potential to fortify WK with GA to improve the beverage without altering its physicochemical characteristics, thereby enhancing its antioxidant capacity. This opens the door for future studies examining the positive health effects of GA when used in a matrix like WK.

## 1. Introduction

Nowadays, consumers are aware of different products available at the supermarket. They are looking for new products with extra health benefits. Therefore, there is a growing demand for food and beverages that can reduce the risk of diseases and improve overall health. Kefir is one of these functional food products gaining popularity. This beverage is produced using kefir grains composed of various fermentative microorganisms, mainly from the genera *Lactobacillus*, *Enterococcus*, *Kazachstania*, *Kluyveromyces*, and *Pichia*. Although kefir has traditionally been produced using dairy sources, it may also be prepared with a sweetened aqueous medium, commonly using brown sugar or other plant-based substrates. These substrates are inoculated with water kefir grains (WKG) to obtain water kefir (WK). In fact, WKG biomass amount limits WK production [1].

In recent years, WK has gained attention for being free of milk proteins such as β-lactoglobulin and casein, which may trigger allergic reactions or lactose intolerance. Consequently, WK represents a suitable alternative for vegans or individuals who avoid dairy products while still benefiting from the functional properties of kefir [2]. WK consumption offers significant health benefits like reduction in plasma cholesterol, prevention of weight gain, and gut–brain axis–regulating properties. Furthermore, by reducing oxidative stress (OS), it has been able to present anti-inflammatory, immunomodulatory, and antioxidant properties [3,4,5,6,7]. Moreover, WK fortification with bioactive compounds may further enhance its functional value.

In recent years, studies have explored kefir fortification with bioactive compounds such as omega-3 fatty acids, vitamin C, kale and spinach, berries, carrots, strawberries, and pomegranate juices [8,9,10,11]. However, most of these studies have focused on dairy kefir, leaving a research gap regarding WK fortification. Despite its potential health benefits, studies on WK fortification remain scarce. Some reports have explored calcium and iron oxide (magnetite) WK fortification as supplements to improve mineral intake by individuals with these deficiencies [12,13]. Nevertheless, the fortification of WK with bioactive molecules, such as gallic acid (GA), has not been explored.

GA is a polyphenolic compound with notable free radical–scavenging capacity. Several studies have shown that GA administration improves cognitive deficits in experimental in vivo models, maintaining the structural integrity of cellular membranes by reducing lipid peroxidation caused by OS. Moreover, it promotes cellular growth and inhibits inflammatory processes [14,15,16]. GA also has very low toxicity; its lethal dose (LD_50_) is 5000 mg/kg according to its safety data sheet, while effective biological activity has been observed at doses between 10 and 1000 µg/kg [17,18]. Concentration effectiveness depends on the matrix and biological system used. However, its direct application is limited due to its acidic nature, hygroscopicity, and light sensitivity. It is necessary to explore new approaches for its incorporation into food matrices, such as WK, and mitigate the OS.

The consumption of WK has been increasing notably in recent years, driven by global trends favoring healthier and functional foods. Due to its simplicity and health-promoting properties, WK represents a promising GA fortified vehicle, potentially enhancing its antioxidant capacity. Pasteurization can alter the physicochemical and functional properties of gallic acid-fortified water kefir due to changes in the stability of phenolic compounds and bioactive metabolites. Furthermore, the heat treatment can reduce the microbial load and affect the metabolic activity of the microorganisms present in the beverage. These changes can influence the antioxidant capacity and bioaccessibility of the compounds during simulated gastrointestinal digestion. Therefore, pasteurization is an important factor in the final quality and functionality of the product. Consequently, we hypothesized that gallic acid fortification would enhance the antioxidant capacity of water kefir and that these effects would be modulated by pasteurization and simulated gastrointestinal digestion.

## 2. Materials and Methods

### 2.1. Water Kefir Grains Activation

WKG were obtained from the Food Engineering Laboratory at the Department of Chemical and Metallurgical Engineering, University of Sonora (Hermosillo, Mexico). Commercial piloncillo, also called panela, was used as a substrate. This sugary solution was prepared by dissolving 9% (*w*/*v*) of piloncillo in purified water. WKG were reactivated by back-slopping at 25 °C for 48 h. This procedure was repeated for two weeks. The grains were subsequently separated by screening and washed during each substrate renewal to ensure complete microbial activation [19]. At the end of this procedure, WKG were washed with tap water and subsequently subjected to the following analyses.

### 2.2. Gallic Acid-Added Water Kefir Beverage Preparations

WK treatments were prepared by dissolving 9% (*w*/*v*) of molasse in purified water and inoculating 9% (*w*/*v*) water kefir grains. Commercial molasses was bought locally (Molienda de Caña Ruiz, Guadalupe de Ures, Sonora, Mexico). GA was obtained from Sigma-Aldrich (St. Louis, MO, USA). The treatments were WK, water kefir with gallic acid [340 mg/L] (WK-GA), pasteurized-WK (pWK), and pWK-GA [340 mg/L]. The GA concentration (340 mg/L) was selected based on previous reports on the incorporation of phenolic compounds into functional beverages and fermented matrices, where similar concentrations have been shown to increase antioxidant capacity without significantly compromising the physicochemical stability of the system. Furthermore, preliminary tests were conducted to verify the compound’s adequate solubility and stability in the fermented matrix, as well as to avoid concentrations that could negatively affect the fermentation process or the beverage’s organoleptic characteristics.

Samples were fermented for 10 h at 25 °C using Erlenmeyer flasks. pWK treatment was heated at 65 °C for 30 min and cooled at 25 °C using an ice bath [20]. The GA addition was performed under aseptic conditions. WKG was separated through sieving analysis. Independent samples (three replicates per sampling time) were taken at 0, 0.5, 1, 2, 3, 4, 6, 8, and 10 h of fermentation, and aliquots were immediately frozen at −80 °C for further analysis.

### 2.3. WKG Dried Biomass and Physicochemical Analyses

WKG biomass (5 g) was weighed in an aluminum tray and dried at 100 °C for 48 h in a laboratory oven [21]. Constant weight was defined as less than 0.001 g between two consecutive weighings. Determinations were performed in triplicate, and results were expressed as mean ± standard deviation. This procedure was used exclusively for quantifying the moisture and dry matter content of the samples. During fermentation time, pH (945.10 AOAC official method), soluble solids (°Brix) (932.14 AOAC official method), and titratable acidity (942.15 AOAC method) were monitored.

### 2.4. Potentially Bioaccessibility and Bioavailability of Gallic Acid-Added Water Kefir Beverages During In Vitro Digestion

The potentially bioaccessibility and digestibility of all treatments were determined using an in vitro gastrointestinal digestion model reported by [22] with modifications. Samples (3 mL) were placed into the mouth of apparently healthy persons for 5 s during the oral phase. This stage was designed to simulate the initial wetting and mixing of the product with saliva during consumption, which is essential for realistically reproducing oral physiological conditions. The selection of this procedure was based on methodologies previously reported in the scientific literature for liquid matrices [22], providing justification and consistency with prior work. Informed consent was obtained from the participant, considering that the procedure involved minimal risk to the volunteer. Samples were transferred to a 50 mL tube with 6 mL physiological solution. The mix was adjusted to pH = 2 with 6 M HCl, and then 6 mL of pepsin was added. Samples were placed in a water bath for 2 h at 37 °C and 80 rpm. The mixture was adjusted to pH = 7 using 1.25 M NaHCO_3_. After that, 5 mL of pancreatin (2 mg/mL) was added to the samples and transferred to a dialysis cellulose membrane (12,000 Da, Sigma, Aldrich). A physiological solution was used outside the membrane. Afterwards, samples were incubated in a water bath for 4 h at 37 °C, and 80 rpm. At the end, samples to evaluate the potentially bioaccessibility were obtained from the inside solution, whereas potentially bioavailability was analyzed through the outside solution of the membrane to measure their antioxidant capacity. “In” and “Out” were used to refer to the internal and external fractions of the dialysis membrane.

### 2.5. Antioxidant Capacity of WK, WK-GA, pWK, pWK-GA, and Their Digests

#### 2.5.1. DPPH^•^ Radical Scavenging Activity

DPPH^•^ (2,2-diphenyl-1-picrylhydrazyl) (1.5 mg) was dissolved in 50 mL of methanol, and the solution absorbance was adjusted to 0.7 ± 0.01 at 515 nm. Subsequently, 200 µL of DPPH^•^ solution was mixed with 20 µL of the samples. The mixtures were incubated in darkness for 30 min, and the absorbance was measured at 515 nm. Results were expressed as DPPH^•^ radical scavenging percentage of inhibition, calculated using Equation (1) [23].

(1) $$\mathrm{DPPH}\;\mathrm{radical}\;\mathrm{scavenging}\;\mathrm{inhibition}\;(\%)\;=\;\frac{Control\;absorbance\;-\;Sample\;absorbance}{Control\;absorbance}\times 100$$

#### 2.5.2. ABTS^•+^ Radical Scavenging Activity

The ABTS^•+^ (2,2′-azino-bis (3-ethylbenzothiazoline-6-sulfonic acid)) radical cation was prepared following the method described by [24]. A volume of 270 µL of the ABTS^•+^ solution was mixed with 20 µL of the samples and incubated for 30 min at room temperature in the dark. Absorbance was read at 734 nm using a 96-well microplate reader (Thermo Fisher Scientific, Waltham, MA, USA). Results were expressed as a percentage of ABTS^•+^ radical scavenging inhibition (Equation (2)).

(2) $$ABTS\;radical\;scavenging\;inhibition\;(\%)\;=\;\frac{Control\;absorbance\;-\;Sample\;absorbance}{Control\;absorbance}\times 100$$

#### 2.5.3. Determination of Half Inhibitory Concentration (IC_50_) of Radicals

Serial dilutions of WK and treatments were used to determine the IC_50_. The calculation was determined using the linear equation. All treatments with >50% of inhibition of DPPH^•^ and ABTS^•+^ radicals were analyzed. The antioxidant capacity was determined according to Section 2.5.1 and Section 2.5.2.

#### 2.5.4. FRAP Assay

The antioxidant capacity was also assessed using the Ferric Reducing Antioxidant Power (FRAP) assay, following the methodology described by [25], with minor modifications. The FRAP reagent was prepared in a 1:1:10 ratio, consisting of 10 mM TPTZ in 40 mM HCl, 20 mM FeCl_3_·6H_2_O, and 0.3 M sodium acetate buffer (pH 3.6). For each measurement, 280 µL of the FRAP reagent was mixed with 20 µL of the sample, and absorbance was measured at 638 nm after 30 min of incubation. A standard calibration curve was constructed using Trolox, and results were expressed as milligrams of Trolox equivalents per milliliter of sample (mg TE/mL).

### 2.6. Statistical Analysis

A completely randomized factorial design was used, where Factor A was the fermentation time (0 or 10 h) and Factor B was the types of beverages (pasteurized beverage, unpasteurized beverage, beverage with added gallic acid, and beverage without gallic acid). Student’s *t*-test was used to compare WK and WK-GA treatments for physicochemical analysis. The data obtained were analyzed using a two-way factorial ANOVA. A 95% confidence level was used (*p* < 0.05). The treatments were performed in triplicate. When significant differences were detected, the means were compared using the multiple means Tukey’s test (≤0.05). The statistical package InfoStat version 2020e (Córdoba, Argentina) was used for all analyses.

## 3. Results and Discussions

### 3.1. Physicochemical Analysis of WK-GA

**Figure 1:**
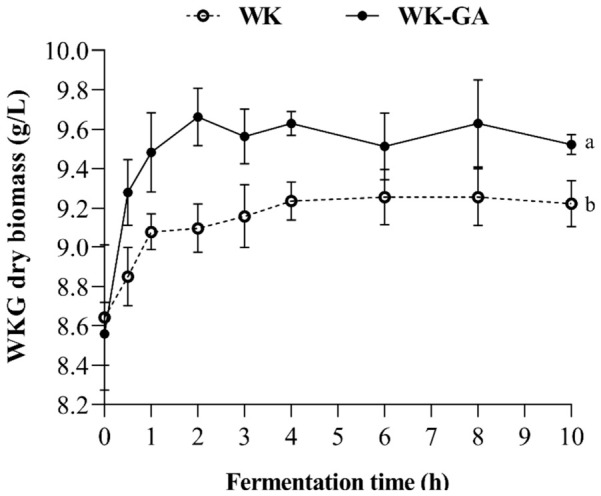
Effect of GA fortification on the dry WKG biomass increase during fermentation time. The data show the mean values ± standard deviation (*n* = 3). Different letters indicate statistically significant differences (*p* < 0.05) at the end of the time.

Figure 1 shows the effect of water kefir fortification with gallic acid during fermentation time through the kinetics of dry water kefir grains biomass growth. WK fortified with 340 mg/L GA showed the maximum biomass increase at the first 2 h of fermentation, 9.66 ± 0.14 g/L. However, the highest biomass increase of WK was at 6 h, 9.25 ± 0.14 g/L. These findings suggest that GA may have played a key role in accelerating the synthesis of WKG biomass at the beginning of the fermentation. Furthermore, it was observed that at the end of fermentation, WK-GA had significantly higher (*p* < 0.05) dry WKG biomass increase than the control. On the other hand, several studies have demonstrated that GA acts as an antimicrobial agent, even acting on LAB involved in the spoilage of red and white meats [26]. However, the concentration used to achieve this antimicrobial effect was 170 times higher than that used to fortify the KA. In addition, Ref. [27] reported that low concentrations of GA may improve the metabolism of BAL, and increasing this concentration above 510 mg/L could alter the cell wall, affecting the viability of the bacteria. On the other hand, Ref. [28] used caffeic, p-coumaric, and ferulic acids in kombucha without significantly affecting its growth.

**Figure 2:**
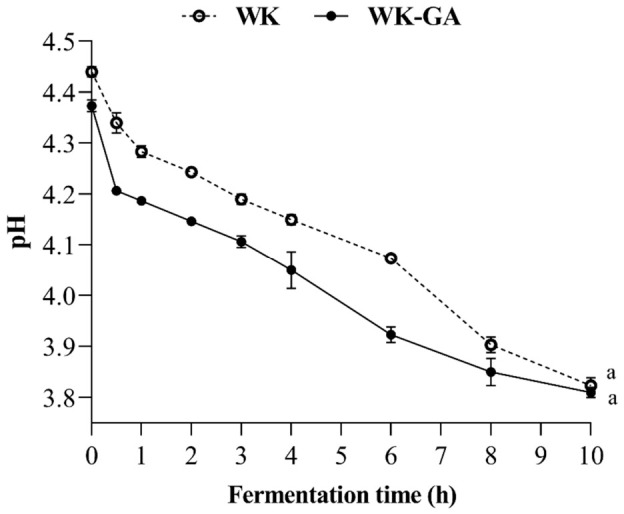
Effect of GA fortification on the pH of the WK beverage during fermentation time. The data show the mean values ± standard deviation (*n* = 3). Different letters indicate statistically significant differences (*p* < 0.05) at the end of the time.

On the other hand, Figure 2 showed that pH decreased as fermentation time progressed. Likewise, during the first two hours, the most pronounced drop in pH was observed. This trend was also evident in the increase in dry biomass. The initial pH of WK fermentation was 4.44 ± 0.01, while WK-GA had 4.37 ± 0.01. At the end of the fermentation process, pH values reached 3.82 ± 0.01 and 3.81 ± 0.01, respectively. No statistically significant differences were observed among the treatments (*p* > 0.05). The reduction in pH after the first 2 h became less abrupt, which may be attributed to the adaptation phase of the KA microbial consortium to the medium during the early hours of fermentation, during which microorganisms consume substrates, release organic acids, and consequently lower pH values. This suggests that after this period, organic acid production occurs gradually. This pattern can also be observed in the increase in titratable acidity.

Ref. [29] reported that fermenting a 6% (*w*/*v*) sucrose solution for 96 h with 150 g/L of WKG resulted in a 3.5 final pH value. Although the markedly longer fermentation period, the final pH was comparable to that observed in the present study after 10 h of fermentation. This similarity may be attributed to the use of molasses as a substrate, which is inherently acidic, exhibiting an initial pH of 4.66 ± 0.02 at a 9% (*p*/*v*) concentration. Ref. [30] reported that fermentation using 5% (*p*/*v*) piloncillo as the substrate at 26 °C for 240 h with 10 g/L of WKG led to a 3.5 final pH value.

**Figure 3:**
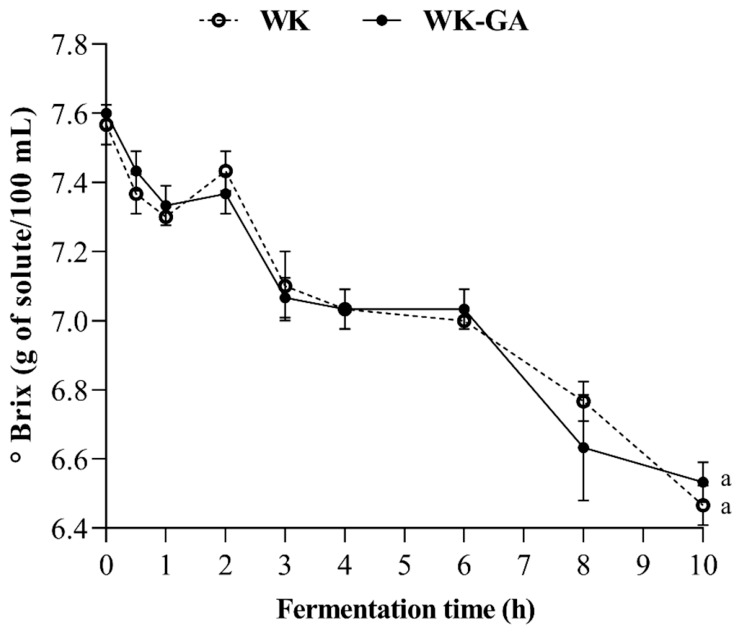
Effect of GA fortification on the °Brix of the WK beverage during fermentation time. The data show the mean values ± standard deviation (*n* = 3). Different letters indicate statistically significant differences (*p* < 0.05) at the end of the time.

The behavior of °Brix during fermentation followed the expected pattern. It began at 7.56 and 7.60 in WK and WK–GA treatments, respectively, and decreased to 6.46 and 6.53 by the end of the process, with no significant differences (*p* > 0.05) between treatments. As observed for pH and dry WK grains biomass, the most pronounced changes occurred during the first three hours. An interesting trend can be seen in T2 (Figure 3), where instead of decreasing, °Brix values increased in both cases, and subsequently declined again at T3. This may indicate that, at the onset of fermentation, microorganisms consume the sucrose present in the molasses; thereafter, glucose and fructose are released into the aqueous medium, along with the production of free dextran by bacteria such as *L. hilgardi*, increasing °Brix. This phenomenon is also reflected in T2 in the observed increase in the biomass of WKG.

**Figure 4:**
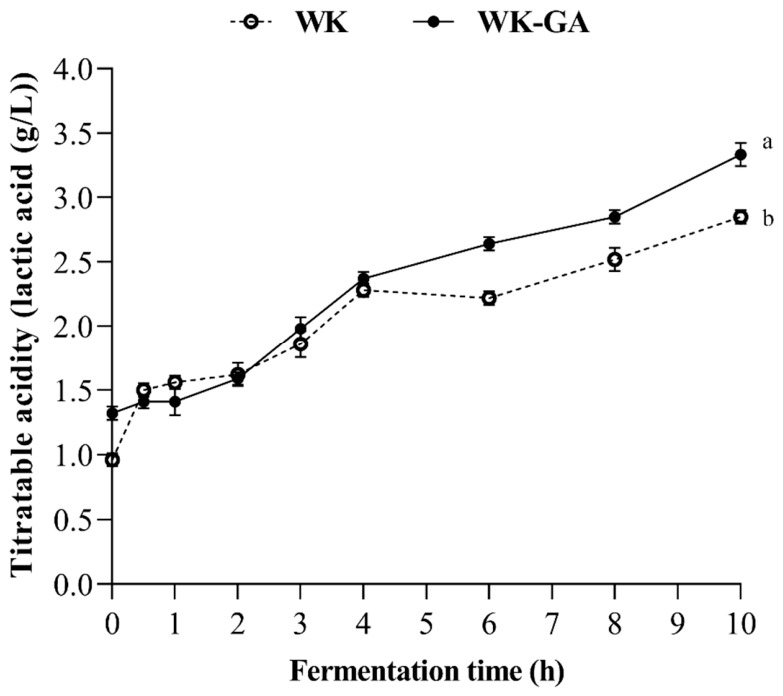
Effect of GA fortification on titratable acidity in WK during fermentation time. The data show the mean values ± standard deviation (*n* = 3). Different letters indicate statistically significant differences (*p* < 0.05) at the end of the time.

On the other hand, Figure 4 shows the kinetics of titratable acidity in WK and WK–GA. The results revealed a clear increasing trend from the beginning to the end of fermentation in both treatments. Initial values were 0.96 g/L lactic acid for WK and 1.32 g/L lactic acid for WK–GA. At the end of the fermentation time, WK-GA fortified had significantly higher (*p* < 0.05) lactic acid concentration than the control, 3.33 g/L and 2.85 g/L lactic acid, respectively. The data indicate that fortifying the beverage with GA not only acidifies the medium at the onset of fermentation and during the first 8 h but also affects titration outcomes. This phenomenon may occur because GA is a weak acid containing a carboxyl group that can be neutralized during the titration process, directly influencing the measured values.

GA fortification has been previously explored in different beverages. Ref. [31] demonstrated that the incorporation of this polyphenol into red wine enhanced the quality and stability of color, where GA interacted with different compounds of wine, such as anthocyanins naturally present in the matrix. Likewise, Ref. [32] investigated the supplementation of drinking water with GA in humans and mice, reporting increased enzymatic activity associated with oxidative stress responses and a concomitant decrease in intracellular reactive oxygen species (ROS) levels. The authors proposed that GA may exert these effects indirectly by modulating cellular signaling pathways involved in oxidative stress regulation.

### 3.2. Kinetics of the Antioxidant Capacity of the Kefir Beverage

The kinetics of antioxidant capacity of WK and WK-GA were evaluated by DPPH^•^, ABTS^•+^, and FRAP.

**Figure 5:**
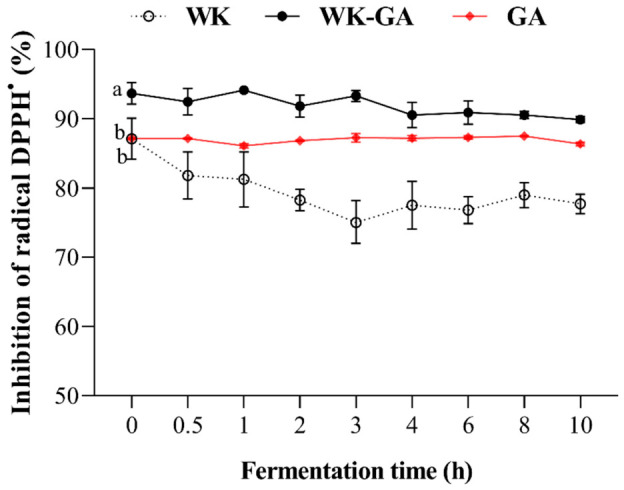
Effect of GA fortification on DPPH^•^ radical scavenging inhibition in WK during fermentation time. Data show mean values ± standard deviation (*n* = 3). Different letters indicate statistically significant differences (*p* < 0.05) between treatments.

Figure 5 shows the behavior of the antioxidant capacity kinetic, determined by the DPPH^•^ assay throughout the fermentation process. WK-GA fortified showed a significantly higher (*p* < 0.05) percentage of DPPH^•^ radical scavenging inhibition at the beginning of the fermentation time, 93.67%, compared to WK, 87.11%. GA was used as a control under the same conditions. The results show that at the beginning of fermentation, GA control was not significantly different (*p* > 0.05) from WK treatment. However, it was observed that the control tends to remain stable over time, while WK tends to decrease its capacity to inhibit the DPPH^•^ radical. This behavior is not observed in WK-GA, suggesting that fortification with GA provides antioxidant stability during the fermentation process. Furthermore, it was observed that WK-GA had a higher percentage of inhibition than the control. This could be due to a possible synergy between the components of the WK substrate that is enhancing the antioxidant capacity of WK-GA after fermentation.

The tendency of WK to decrease antioxidant capacity during fermentation, starting with an inhibition of the DPPH^•^ radical of 87.11% and ending with 77.72%, was also observed in studies conducted by [33]. These authors reported that fermentation with carob sorbet and WKG decreased over time, likely because the enzymes present in the WKG microorganisms transform phenolic compounds into the medium. Likewise, it depends on the substrate used for fermentation, the way that the microorganisms behave, and whether they metabolize the compounds present in the beverage [34]. Add GA to WK during fermentation; this phenol remains present and helps to sustain the antioxidant capacity for the 10-h fermentation period.

**Figure 6:**
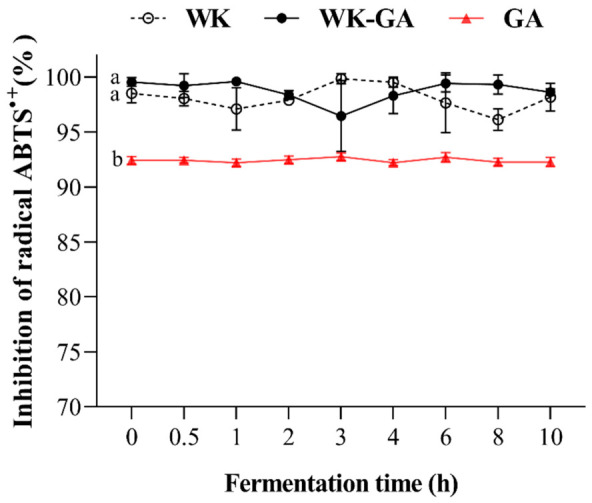
Effect of GA fortification on ABTS^•+^ radical scavenging inhibition in WK during fermentation time. Data show mean values ± standard deviation (*n* = 3). Different letters indicate statistically significant differences (*p* < 0.05) at the end of the time.

Furthermore, the results of the antioxidant capacity assessment by inhibiting the ABTS^•+^ radical scavenging showed that WK fortified with GA had 99.55% radical inhibition and WK 98.51% at the beginning of the fermentation, while the GA control started with 92.24% (Figure 6). Statistical analysis revealed that WK-GA and WK did not differ significantly (*p* > 0.05) from each other at that fermentation time. Additionally, the antioxidant capacity of the beverages varied during fermentation. This suggests that molecules inherent to the molasses (substrate) are being released and metabolized during this process, which may explain the variability in the results. However, at the end of fermentation, both WK-GA and WK inhibited more than 98% of the radicals.

The results showed that WK and WK-GA exhibited the same trend in ABTS^•+^ radical scavenging inhibition kinetics, suggesting that the substrate’s inherent nature may be contributing to the beverage’s antioxidant capacity. This could be due to the ABTS^•+^ technique’s affinities, as it reacts more readily with molecules such as phenols and flavonoids. A variety of these compounds have also been reported in molasses, including chlorogenic acid, caffeic acid, synaptic acid, syringic acid, vanillin, homoorientin, orientin, vitexin, swertisin, diosmin, apigenin, tricin, and diosmetin [35]. These may be enhancing the antioxidant capacity of the substrate and the beverages. On the other hand, in a study conducted by [36], they showed that the phenolic components, carotenoids, and flavonoids in WK are closely related to the antioxidant capacity of the beverage.

**Figure 7:**
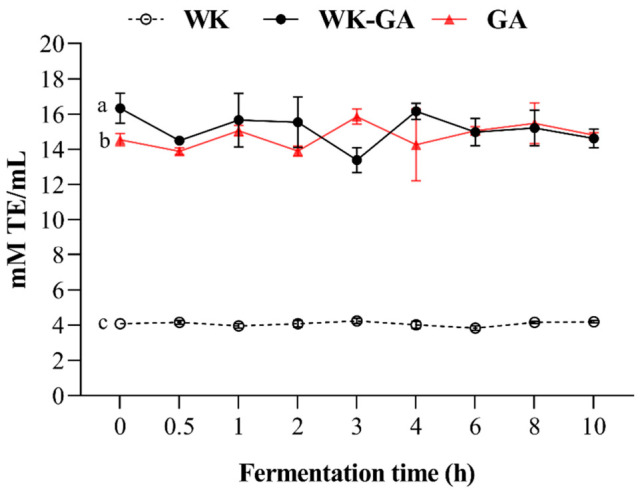
Effect of GA fortification on FRAP assay in WK during fermentation time. Data show mean values ± standard deviation (*n* = 3). Different letters indicate statistically significant differences (*p* < 0.05) at the end of the time.

Figure 7 shows the antioxidant capacity determined by the FRAP assay. WK-GA’s iron-reducing capacity is due to its fortification with this polyphenol. The results show a significant difference from the start of fermentation compared to WK. The increase in antioxidant capacity through the FRAP assay was from 4.07 mM TE/mL for WK to 16.33 for WK-GA, at the beginning of the fermentation. Likewise, as fermentation kinetics progressed, the dynamics of WK-GA changed. This suggests that the behavior of the microorganisms and their metabolism varied, thereby altering the values obtained in the antioxidant assays. At 3 h fermentation time, the iron-inhibiting capacity of WK-GA decreased to 13.39 mM TE/mL. However, in the next hour, it increased to 16.16 mM TE/mL. Interestingly, at 6 h, it dropped to 15 mM TE/mL and maintained the trend until the end of fermentation.

#### Antioxidant Capacity and IC_50_ of the Kefir Beverages and Their Digests

While in vitro digestion is a valuable tool for studying digestive processes and evaluating nutrient bioavailability, it has some limitations. For example, it does not fully replicate the physiological conditions of the human digestive tract, including the interaction between different types of food, the gut microbiota, and the variability in digestive enzyme production. However, it offers several advantages, such as the ability to precisely control and manipulate experimental conditions, allowing for the study of the specific effects of different foods and nutrients. Furthermore, it is a practical methodology, and finally, it facilitates large-scale studies and the obtaining of reproducible results, which is crucial for the development of new food products and supplements.

WK samples from all treatments were selected at 10 h fermentation time. All beverages underwent in vitro digestion. The WK, pWK, WK-GA, and pWK-GA beverages served as controls without in vitro digestion. The digests were subjected to DPPH^•^, ABTS^•+^, and FRAP assays. Tests were performed on undigested beverages and their digests, both inside and outside the dialysis membrane, to assess the potential bioaccessibility and bioavailability of treated beverages. However, the interaction between antioxidants and other food components, such as carbohydrates and fats, can influence their bioavailability.

**Figure 8:**
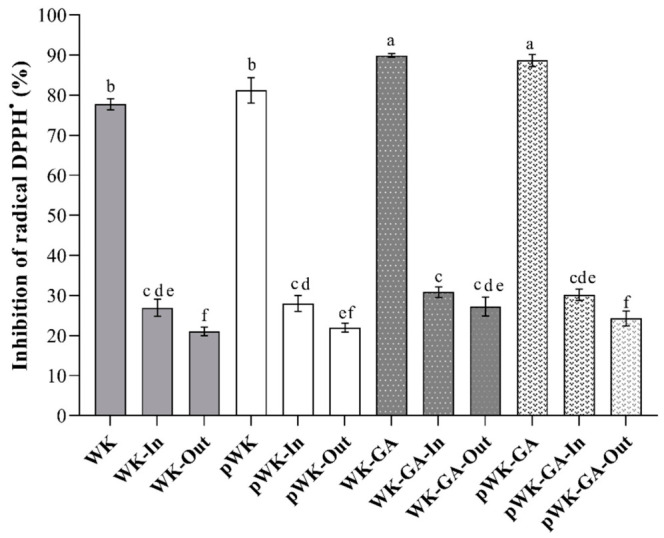
DPPH^•^ radical scavenging inhibition of the bioaccessibility and bioavailability of pasteurized and unpasteurized WK fractions. Data show mean values ± standard deviation (*n* = 3). Different letters indicate statistically significant differences (*p* < 0.05).

Results obtained from the DPPH^•^ radical scavenging inhibition indicated that WK fortified with GA, unpasteurized and pasteurized treatments presented the highest values, 90 and 89.5 of inhibition, respectively. In fact, there was no significant difference (*p* > 0.05) between them. However, there was a significant difference (*p* < 0.05) between the beverages with and without undigested GA, as shown in Figure 8.

The antioxidant capacity decreased significantly in the digests that represent bioaccessibility regardless of pasteurization and GA fortification. Similarly, the digests outside the dialysis membrane, representing bioavailability, were statistically similar except for WK-GA-Out, which was only equal to pWK-Out. This suggests that GA fortification in the beverage may be contributing to the inhibition of the DPPH^•^ radical in the bioavailable fraction of the beverage. Furthermore, the DPPH^•^ technique performs better at acidic pH levels, which could be affecting the measurements, given that the final pH of the digests was approximately 7, while in the undigested beverages (WK, pWK, WK-GA, and pWK-GA), the pH was 3.81, 3.89, 3.82, and 3.86, respectively. However, the digests representing bioavailability still managed to inhibit the DPPH^•^ radical, where WK-GA-Out showed the greatest inhibition at 27%.

On the other hand, the DPPH^•^ technique has a higher affinity for lipophilic molecules such as tocopherols and carotenoids, suggesting that fine structures are likely present before digestion, but these lose activity after digestion [9]. Studies by [37] have shown that this pattern occurs when the DPPH^•^ antioxidant capacity of water-based kefir decreased, suggesting that bioactive molecules could also be lost due to pH. Therefore, this research uses three different methods to measure antioxidant capacity and obtain a broad overview of the behavior of WK and its treatments.

**Figure 9:**
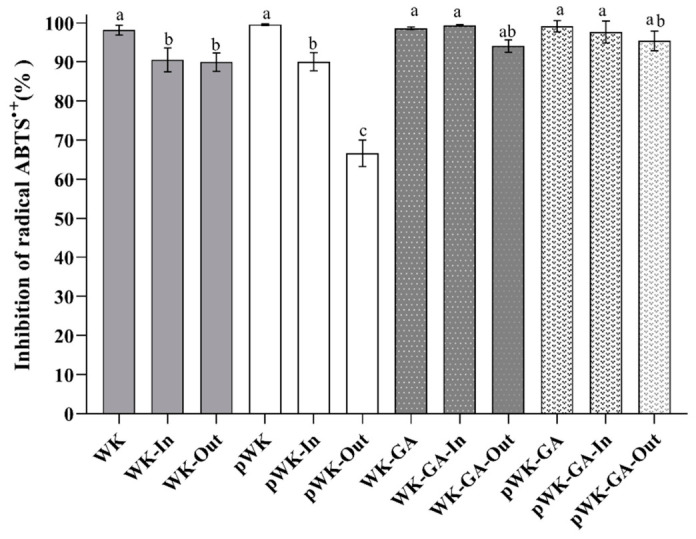
ABTS^•+^. Comparison of the antioxidant capacity of WK with GA, pasteurized, and their digests In and Out of the dialysis membrane. Data show mean values ± standard deviation (*n* = 3). Different letters indicate statistically significant differences (*p* < 0.05).

Figure 9 shows comparisons of the beverages and digests with respect to ABTS^•+^ radical inhibition. The results showed no significant difference between WK, pWK, WK-GA, and pWK-GA, with over 98% inhibition achieved in all cases. Similarly, the data showed no significant difference in the digests of WK-GA-In, WK-GA-Out, WK-GA-In, and pWK-GA-Out, maintaining a radical percentage greater than 94%. Likewise, the bioaccessible and bioavailable fractions in the WK digests (WK-In and WK-Out) showed 90% and 89% radical inhibition, respectively, with no significant differences between them. However, the results showed that the digests of the pasteurized beverage without GA fortification significantly decreased the radical inhibition percentage. However, a decrease in antioxidant capacity was observed in the bioavailable fraction (pWK-Out), which maintained radical inhibition above 66%. Interestingly, fortification of WK with GA, regardless of pasteurization, maintained inhibition above 94% in the bioavailable fraction. This suggests that the GA in the beverage is contributing to the stability of WK antioxidant capacity. Furthermore, the affinity of the ABTS^•+^ radical for molecules such as phenols and flavonoids may be contributing to the antioxidant capacity. Given that the WK produced in this study is molasses-based, it also contains, in smaller proportions, molecules such as caffeic and syringic acids, as well as flavonoids like orientin and diosmetin [35]. These molecules and ABTS^•+^ technique tolerance to acidic and slightly basic pH levels may be influencing the results obtained in this study.

This behavior was observed in a recent WK study with a complex substrate supplemented with pistachio extract [37]. In the ABTS^•+^ assay, the antioxidant capacity was maintained after digestion. This is explained by the fact that during digestion, components with an affinity for the ABTS^•+^ radical are likely released, or the pH of the assay may facilitate the reaction, thus maintaining antioxidant capacity. These results are consistent with those obtained in this investigation, where WK-GA and its digests maintained the ability to inhibit the ABTS^•+^ radical even after in vitro digestion.

**Figure 10:**
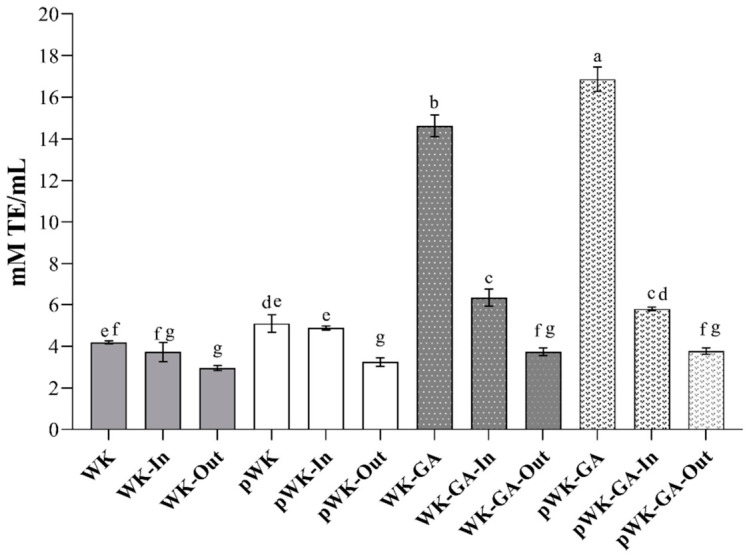
FRAP. Comparison of the antioxidant capacity of WK with GA, pasteurized, and their digests In and Out of the dialysis membrane. Data show mean values ± standard deviation (*n* = 3). Different letters indicate statistically significant differences (*p* < 0.05).

Finally, the iron-reducing capacity of the beverages and digests is shown in Figure 10. The results showed that pasteurization had no significant effect on the iron-reducing capacity of WK and pWK (4.19 and 5.09 mM TE/mL, respectively). Likewise, the bioavailable fractions of WK and pWK were statistically equal, with values of 2.9 and 3.209 mM TE/mL, respectively. However, WK-GA had a lower iron-reducing capacity than pWK-GA, indicating that reducing molecules are likely formed during the pasteurization process.

It is important to note that in the WK-GA beverage, fortification was carried out from the beginning of fermentation, while in pWK-GA, heat treatment was performed after 10 h of fermentation, followed by fortification with GA. This may be related to the increased reducing power of pWK-GA because the GA was not in contact with microorganisms or other processes during fermentation. Likewise, it is likely that the GA is structurally intact, enhancing its electron donation capacity. This would not be reflected in the DPPH^•^ and ABTS^•+^ studies because, in both cases, the mechanism can be attributed to single electron transfer (SET) and hydrogen atom transfer (HAT), whereas in FRAP, it is exclusive to the SET mechanism. Furthermore, the data showed that the bioavailable fractions WK-GA-Out and pWK-GA-Out are statistically equal to WK, demonstrating that GA fortification is achieving this effect. Interestingly, the bioavailable fractions of all treatments were significantly the same.

In addition, the IC_50_ values (Table 1) of all treatments with >50% inhibition of ABTS^•+^ and DPPH^•^ radicals were analyzed. The results show that all GA-fortified beverages, regardless of pasteurization, achieved a lower IC_50_ than unfortified beverages. This indicates that GA has a positive effect on antioxidant capacity as measured by ABTS^•+^ and DPPH^•^. This behavior was also observed in WK-GA-Out and pWK-GA-Out dialysates measured by ABTS compared to WK-Out and pWK-GA-Out. This data provides important information on the potential role of GA in fortifying the beverage even after in vitro digestion.

The antioxidant properties of kefir-based beverages depend on the fermentation conditions, the type of substrate, and the source of kefir grains. Studies conducted by [9] demonstrated that milk-based kefir smoothies fortified with kale and spinach increased the beverage’s antioxidant capacity. They also indicated that after in vitro digestion, FRAP values decreased in the kale kefir while they increased in the spinach kefir, although not significantly. This is due to the components of these vegetables, which contain molecules such as chlorogenic, caffeic, and gallic acids, as well as carotenoids and flavonoids. In our studies with WK, pWK, WK-GA, and pWK-GA, the iron-reducing capacity decreased compared to the digests. However, it should be noted that the fermentation characteristics are different, primarily influenced by the substrate and whether it is used in a water-based kefir beverage. On the other hand, Ref. [38] reported a decrease in FRAP values in a milk-based kefir after in vitro digestion. In this case, the decrease was related to the loss of peptides during digestion. In our study, this can be extrapolated to a loss of molecules capable of reducing iron, in addition to influencing the final pH of the digests to approximately 7. This is not necessarily negative, since fortification with GA could provide benefits by activating signaling pathways to produce pro-oxidant enzymes and anti-inflammatory molecules [39].

When analyzing the results of gallic acid (GA) fortification, it is observed that the added value of this intervention is primarily manifested in the bioaccessible fraction. It is crucial to highlight that, in this fraction, antioxidant activity tends to decrease below the saturation level, implying that not all antioxidant compounds behave linearly with respect to their concentration. This decrease suggests that, upon reaching a specific threshold, the antioxidant capacity of the fortified compounds may be limited, affecting the interpretation of the differences between the analyzed samples. Furthermore, adequately assessing these differences in antioxidant activity is fundamental to understanding the true impact of GA fortification on antioxidant bioavailability. Therefore, it is recommended that future research delve deeper into the interactions between GA and other food components in the bioaccessible fraction, as well as their influence on health and the potential for developing fortified food products. These findings underscore the importance of considering the bioaccessible fraction as a key factor in evaluating the effectiveness of antioxidant fortification.

On the other hand, the results obtained using the DPPH, ABTS, and FRAP assays allowed for a comprehensive evaluation of the antioxidant capacity of the samples, considering different mechanisms of action related to electron transfer and free radical neutralization. The combined use of these methods provided a functional approximation of the effect of GA fortification and processing conditions on the antioxidant properties of WK before and after in vitro digestion.

However, although these assays are widely used to estimate the total antioxidant capacity of food matrices, their results represent an overall response and do not allow for the specific identification of the compounds responsible for the observed activity or the complete establishment of the biochemical mechanisms involved. In complex fermented systems, such as WK, antioxidant activity can be influenced by multiple components, including phenolic compounds, metabolites generated during fermentation, bioactive peptides, and potential products derived from interactions between matrix constituents.

Therefore, the mechanistic interpretation of the results should be approached with caution. Complementary studies based on chemical characterization techniques, such as HPLC or LC-MS, could help to identify and quantify the bioactive compounds present, as well as to establish a more precise relationship between the chemical composition and the observed antioxidant capacity. These analyses were not included in the present study due to limitations related to the availability of specialized analytical infrastructure, experimental time, and the methodological scope of the work. The main objective of this research was to functionally evaluate the effects of gallic acid fortification and processing on the physicochemical properties and antioxidant capacity of WK in an in vitro digestion model. Therefore, the use of widely accepted global antioxidant assays was prioritized to establish an initial characterization of the functional behavior of the samples. Consequently, future studies will consider the application of methodologies such as HPLC and LC-MS to further the mechanistic interpretation of the observed antioxidant activity and establish more precise correlations between chemical composition and biological functionality.

Furthermore, although the observed changes in antioxidant capacity and physicochemical properties could be related to metabolic transformations associated with microbial activity during fermentation, this study did not include specific analyses of the microbial community or detailed characterization of metabolites. Therefore, these interpretations are considered inferential. Future studies based on microbial sequencing and metabolomics will allow for the establishment of more precise mechanistic relationships between microbial activity and the observed functional changes.

## 4. Conclusions

This research evaluated the effect of GA fortification on some physicochemical properties and the antioxidant capacity of water kefir (WK) before and after in vitro digestion. The results showed that GA addition promoted biomass growth and contributed to maintaining higher antioxidant levels during the fermentation process, particularly in free radical inhibition and reducing capacity assays. Pasteurization had a limited effect on the antioxidant capacity of the digested fractions, depending on the analytical method used. However, the results obtained correspond only to in vitro evaluations. Therefore, future studies should include chemical characterization analyses and in vivo models to more accurately understand the functional impact of GA fortification in fermented beverages.

## Figures and Tables

**Table 1:** IC_50_ of WK and treatments on antioxidant capacity.

	WK	pWK	WK-GA	pWK-GA	WK-In	pWK-In	WK-GA-In	pWK-GA-In	WK-Out	pWK-Out	WK-GA-Out	pWK-GA-Out
ABTS^•+^ (µL/mL)	0.0104	0.0098	0.0022	0.0024	0.0365	0.0416	0.0211	0.0244	0.0370	0.0633	0.0280	0.0257
DPPH^•^ (µL/mL)	0.0223	0.0133	0.0022	0.0033	ND	ND	ND	ND	ND	ND	ND	ND

ND: Not detectable because even 50% inhibition was not achieved in these samples.

## Data Availability

The original contributions of data presented in this research are included in the article. Further inquiries can be directed to the corresponding authors.

## Abbreviations

The following abbreviations are used in this manuscript:

AAPH	2,2-Azobis(2-methylpropionamidine) dihydrochloride
ABTS^•+^	2,2-azino-bis-(3-ethylbenzothiazoline-6-sulfonic acid
DPPH^•^	2,2-diphenyl-1-picrylhydrazyl
FRAP	Ferric reducing antioxidant power
GA	Gallic acid
OS	Oxidative stress
TPTZ	2,4,6-Tripridil-s-triazine
Trolox	6-hydroxy-2,5,7,8-tetramethylchroman-2-carboxylic acid
WK	Water kefir
WKG	Water kefir grains
WK-GA	GA-fortified WK
pWK	Pasteurized water kefir
pWK-GA	Pasteurized water kefir with gallic acid
